# A review on the chemical constituents and pharmacological efficacies of *Lindera aggregata* (Sims) Kosterm

**DOI:** 10.3389/fnut.2022.1071276

**Published:** 2023-01-16

**Authors:** Yangbin Lv, Yanfang Zou, Xindan Zhang, Bingrui Liu, Xin Peng, Chu Chu

**Affiliations:** ^1^College of Pharmaceutical Sciences, Zhejiang University of Technology, Hangzhou, China; ^2^School of Public Health, North China University of Science and Technology, Tangshan, China; ^3^Ningbo Municipal Hospital of Traditional Chinese Medicine, Affiliated Hospital of Zhejiang Chinese Medical University, Ningbo, China

**Keywords:** *Lindera aggregata* (Sims) Kosterm., chemical constituents, pharmacological activity, nutrient, Chinese medicines

## Abstract

*Lindera aggregata* (Sims) Kosterm. (*L. aggregata*), which belongs to the genus *Lindera* in the family Lauraceae, is widely distributed in Asia and the temperate, tropical regions of North America. Its roots and leaves have been used for thousands of years as traditional Chinese medicine and/or functional food. To further explore its underlying nutritional value, this review provided a comprehensive insight into chemical constituents and pharmacological effects on *L. aggregata*. The phytochemical investigation of different parts of *L. aggregata* led to the identification of up to 349 components belonging to sesquiterpenoids, alkaloids, flavonoids, essential oils, and other compounds. Among them, sesquiterpenoids, flavonoids, and alkaloids are assessed as representative active ingredients of *L. aggregata*. A wide variety of pharmacological effects of *L. aggregata*, such as anti-hyperlipidemic, anti-tumor, anti-inflammatory, analgesic, and anti-oxidant, have been proved *in vitro* and *in vivo*. In summary, this review aims to provide a scientific basis and reference for further research and utilization of *L. aggregata* and lay the foundation for developing functional foods with potential active ingredients for the prevention and management of related diseases.

## 1. Introduction

The genus *Lindera* is a member of the Lauraceae family, containing approximately 100 species and widely distributed in tropical, subtropical, and temperate zones of Asia and Midwestern America (1). *Lindera* plants are widely used in traditional medicine, of which *Lindera aggregata* (Sims) Kosterm. [*Lindera strychnifolia* (Siebold et Zucc.) Fern.-VIll] is a representative one (2). It is known chiefly as “Wu-Yao,” a folk plant in China, which mainly grows in the eastern, central, southern, and southwestern parts of China, such as Zhejiang, Jiangxi, and Hunan provinces (3). The roots and leaves of *L. aggregata* are suggested as medicinal and edible parts. More specifically, as an edible plant, the tender leaves of *L. aggregata* are consumed as a functional tea or dietary supplement for its health-promoting benefits, such as anti-hepatic injury and lipid-lowing activity (4). Also, as an herbal medicine, it has been reported that the roots of *L. aggregata* (LA-R) could treat the diseases of gastrointestinal tract, metabolism, inflammation, urinary system, etc. LA-R is included in 24 formulae in Chinese Pharmacopoeia (2020ed) (1, 5). In addition, the leaves of *L. aggregata* (LA-L) are beneficial for treating mastitis, acute cellulitis, carbuncles, and rheumatoid arthritis (6, 7). The health benefits of *L. aggregata* are mainly attributed to its diverse bioactive constituents, such as sesquiterpenoids, alkaloids, flavonoids, and essential oils. And these bioactive components contribute to its multiple health functions, including hepatoprotective, anti-inflammatory, anti-virus, anti-bacterial, anti-tumor, anti-oxidant efficacies, etc. (8).

Although the chemical composition and biological activity of *L. aggregata* have been analyzed extensively, existing reviews fail to offer a comprehensive and systematic overview, owing to the complexity of the natural medicinal plants and the advance of novel analytical techniques. A detailed overview of the phytochemistry and medicinal properties was carried out for the whole genus of *Lindera* plants in 2016 (1). Two reviews on the new progress in phytochemistry and biological activities of *L. aggregate* has been reviewed in the last 5 years (3, 8), but they were still found incomplete in both the summary of chemical compounds and the in-depth discussion on pharmacological mechanism. With the increasing focus on *L. aggregata*, a systematic review is urgently needed on the recent progress in the discovery of components, together with pharmacological investigations in different parts of *L. aggregate*, aiming to inspire the interest of phytochemists and promote the development and utilization of this valuable plant for various remedies.

## 2. Chemical compounds

**FIGURE 1:**
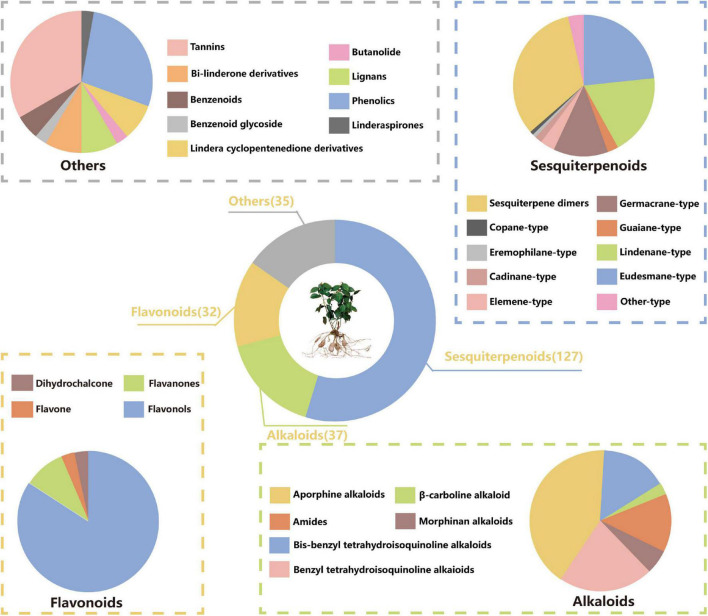
Proportion of chemical compounds in *L. aggregata.*

Many studies have been conducted to explore the phytoconstituents from *L. aggregata*. Up to now, 349 compounds have been isolated and identified from *L. aggregata*. Based on the chemical structures, these compounds can be divided into six groups: 127 sesquiterpenoids (including dimeric and trimeric sesquiterpenoids), 37 alkaloids (including amides), 32 flavonoids, 35 other components, and 118 essential oils (except those sesquiterpenoids already mentioned before). The information about different types of compounds is summarized below in Figure 1.

### 2.1. Sesquiterpenoids

**FIGURE 2:**
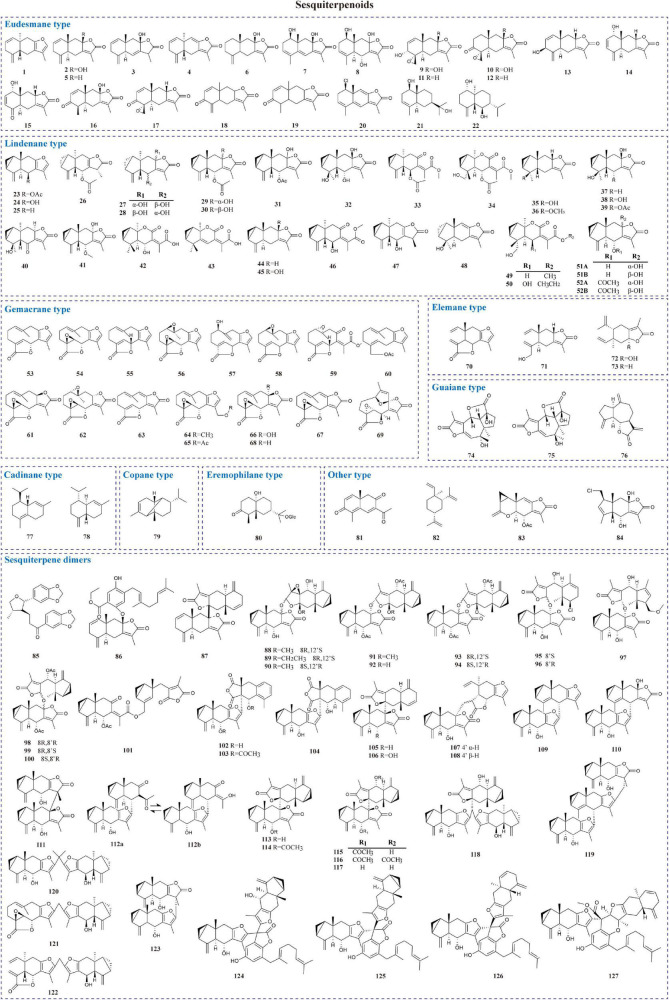
Chemical structures of sesquiterpenoids in *L. aggregata.*

Sesquiterpenoids possess notable biological activities and are the primary active ingredients in *L. aggregata*. They are constructed by three isoprene units (15 carbon atoms) with chains, rings, and other diverse skeletons of structures. To date, 127 sesquiterpenoids have been reported, mostly isolated from roots and a few obtained from whole plants and leaves. As shown in Table 1 and Figure 2, modern phytochemistry studies reveal that the sesquiterpenoids isolated from *L. aggregata* are mainly featured as monomer-, dimer-, and hybrid forms. The sesquiterpenoid monomers include eudesmane- (1–22), lindenane- (23–52), germacrane- (53–69), elemane- (70–73), guaiane- (74–76), cadinane- (77–78), copane- (79), eremophilane- (80), and other types (81–84) (9–27). So far, the sesquiterpenes of *L. aggregata* account for more than half of this kind of compounds in the whole genus *Lindera*, especially the eudesmane-, lindenane-, and germacrane-types, which were almost all found in *L. aggregata* (1). Thereinto, linderagalactone A (84) is a halogenated sesquiterpene lactone possessing a unique rearranged carbon skeleton (13). Besides that, eudesmanes exhibit liver protection (3, 8) (13), anti-cancer (18, 19) (15), anti-fibrotic (1, 14) (9), and anti-inflammatory properties (11, 12) (14); lindenanes exhibit anti-inflammatory properties (36, 37) and anti-oxidant activity (23) (20, 27); germacranes (53, 54) (13) exhibit liver protection activity. Furthermore, in elemanes, the representative compound isolinderalactone (ILL) (70) has significant anti-tumor activity (28–31). And compound 83 shows anti-inflammatory properties (16).

**TABLE 1:** Sesquiterpenoids isolated from *L. aggregata.*

Subtype	No.	Constituents	Molecular formula	Parts	References
Eudesmane-type	1	Lindestrene	C_15_H_18_O	Roots	(9, 24)
	2	Hydroxylindestrenolide	C_15_H_18_O_3_	Roots	(10, 24)
	3	8-hydroxylindestenolide	C_15_H_18_O_3_	Roots	(11)
	4	Dehydrolindestrenolide	C_15_H_16_O_2_	Roots	(12, 24)
	5	Lindestrenolide	C_15_H_18_O_2_	Roots	(9, 24)
	6	Atractylenolide III	C_15_H_20_O_3_	Roots	(13)
	7	Linderagalactone D	C_15_H_18_O_4_	Roots	(13)
	8	Linderagalactone E	C_15_H_20_O_5_	Roots	(13)
	9	Linderolide A	C_15_H_18_O_5_	Roots	(14)
	10	Linderolide B	C_15_H_18_O_5_	Roots	(14)
	11	Linderolide C	C_15_H_18_O_4_	Roots	(14)
	12	Linderolide D	C_15_H_18_O_4_	Roots	(14)
	13	Linderolide E	C_15_H_18_O_3_	Roots	(14)
	14	Linderolide G	C_15_H_18_O_3_	Roots	(9)
	15	Linderolide H	C_14_H_16_O_4_	Roots	(9)
	16	Linderolide I	C_15_H_18_O_4_	Roots	(9)
	17	Linderolide J	C_15_H_16_O_5_	Roots	(9)
	18	3-oxo-5α*H*,8β*H*-eudesma-1,4(15),7(11)-trien-8,12-olide	C_15_H_16_O_3_	Roots	(15)
	19	3-oxo-4,5α*H*,8β*H*-eudesma-1,7(11)-dien-8,12-olide	C_15_H_18_O_3_	Roots	(15)
	20	Linderaggredin A	C_15_H_15_ClO_2_	Whole plants	(16)
	21	3-eudesmene-1β,11-diol	C_15_H_26_O_2_	Roots	(13)
	22	*ent*-4(15)-eudesmene-1β,6α-diol	C_15_H_26_O_2_	Roots	(10)
Lindenane-type	23	Lindenenyl acetate	C_17_H_20_O_3_	Roots	(27)
	24	Lindenenol (linderene)	C_15_H_18_O_2_	Roots, Leaves	(9, 12, 17–19)
	25	Lindenene	C_15_H_18_O	Roots	(20)
	26	Lindenanolide A	C_17_H_20_O_4_	Roots	(17)
	27	Lindenanolide B1	C_15_H_18_O_4_	Roots	(17)
	28	Lindenanolide B2	C_15_H_18_O_4_	Roots	(17)
	29	6α-acetyl-lindenanolide B1	C_17_H_20_O_5_	Roots	(17)
	30	6α-acetyl-lindenanolide B2	C_17_H_20_O_5_	Roots	(17)
	31	Linderanlide F	C_17_H_20_O_5_	Roots	(10)
	32	Linderolide K	C_15_H_20_O_5_	Roots	(9)
	33	Linderolide L	C_18_H_22_O_5_	Roots	(9)
	34	Linderolide M	C_18_H_24_O_7_	Roots	(9)
	35	Linderolide N	C_15_H_20_O_3_	Roots	(20)
	36	Linderolide O	C_16_H_22_O_3_	Roots	(20)
	37	Linderolide P	C_15_H_20_O_4_	Roots	(20)
	38	Linderolide Q	C_15_H_20_O_5_	Roots	(20)
	39	Linderolide R	C_17_H_22_O_6_	Roots	(20)
	40	Linderolide S	C_15_H_18_O_4_	Roots	(20)
	41	Linderolide T	C_16_H_20_O_4_	Roots	(20)
	42	Linderagalactone B	C_15_H_20_O_5_	Roots	(13)
	43	Linderagalactone C	C_15_H_18_O_4_	Roots	(13)
	44	Shizukanolide	C_15_H_18_O_2_	Roots	(9)
	45	Chloranthalactone D	C_15_H_18_O_3_	Roots	(9)
	46	Linderolide U	C_16_H_20_O_4_	Roots	(12, 26)
	47	Linderolide V	C_15_H_18_O_3_	Roots	(26)
	48	Linderaggredin B	C_15_H_18_O_3_	Whole plants	(16)
	49	Linderanolide G	C_16_H_22_O_6_	Roots	(14, 23)
	50	Strychnilactone	C_17_H_24_O_6_	Roots	(21, 22)
	51	Strychnistenolide	C_15_H_18_O_4_	Roots	(13, 20, 21)
	52	Strychnistenolide 6-*O*-acetate	C_17_H_20_O_5_	Roots	(20, 21)
Germacrane-type	53	Linderalactone	C_15_H_16_O_3_	Roots	(24)
	54	Linderane	C_15_H_16_O_4_	Roots	(9–14, 17, 19, 20, 24)
	55	Neolinderalactone	C_15_H_16_O_3_	Roots	(13)
	56	(+)-linderadine	C_15_H_16_O_5_	Roots	(10)
	57	Parvigemone	C_15_H_16_O_4_	Roots	(20)
	58	Pseudneolinderanec	C_15_H_16_O_4_	Roots	(20)
	59	Neolindenenonelactone	C_16_H_18_O_6_	Roots	(11)
	60	Neosericenyl acetate	C_15_H_20_O_3_	Roots	(24)
	61	Linderanlide A	C_15_H_16_O_5_	Roots	(10)
	62	Linderanlide B	C_15_H_16_O_6_	Roots	(10)
	63	Linderanlide C	C_15_H_14_O_4_	Roots	(10)
	64	Linderanlide D	C_16_H_18_O_5_	Roots	(10)
	65	Linderanlide E	C_17_H_18_O_6_	Roots	(10)
	66	Linderanine A	C_15_H_16_O_6_	Roots	(10)
	67	Linderanine B	C_15_H_14_O_5_	Roots	(10)
	68	Linderanine C	C_15_H_16_O_5_	Roots	(10)
	69	Linderoline	C_15_H_14_O_6_	Roots	(10)
Elemane-type	70	Isolinderalactone	C_15_H_16_O_3_	Roots	(24)
	71	Linderolide F	C_15_H_20_O_3_	Roots	(14)
	72	Hydroxyisogermafurenolide	C_15_H_20_O_3_	Roots	(13)
	73	Isogermafurenolide	C_15_H_20_O_2_	Roots	(24)
Gualane-type	74	Lindenanolide C	C_16_H_18_O_6_	Roots	(17)
	75	Lindenanolide D	C_16_H_18_O_6_	Roots	(17)
	76	Dehydrocostuslactone	C_14_H_18_O_2_	Roots	(10)
Cadinane-type	77	(+)-cadinene	C_15_H_24_	Mesocarp and seed	(24)
	78	(±)-cadina-4,10 (15)-diene	C_15_H_24_	Mesocarp and seed	(24)
Copane-type	79	Ylangene	C_15_H_24_	Roots	(24)
Eremophilane-type	80	10,11-dihydroxyeremophilan-3-one 11-*O*-β-D-glucopyranoside	C_21_H_36_O_8_	Roots	(25)
Other type	81	Linderaggredin D	C_14_H_14_O_3_	Whole plants	(16)
	82	β-elemene	C_15_H_24_	Roots	(24)
	83	Linderaggredin C	C_17_H_18_O_5_	Whole plants	(16)
	84	Linderagalactone A	C_15_H_19_ClO_4_	Roots	(13)
Sesquiterpene dimers	85	Linderin A	C_22_H_22_O_6_	Roots	(19)
	86	Linderin B	C_34_H_42_O_6_	Roots	(19)
	87	Bilindestenolide	C_30_H_34_O_4_	Roots	(24)
	88	Linderaggrenolide A	C_31_H_38_O_8_	Roots	(32)
	89	Linderaggrenolide B	C_32_H_40_O_8_	Roots	(32)
	90	Linderaggrenolide C	C_31_H_38_O_8_	Roots	(32)
	91	Linderaggrenolide D	C_35_H_42_O_9_	Roots	(32)
	92	Linderaggrenolide E	C_34_H_40_O_9_	Roots	(32)
	93	Linderaggrenolide F	C_35_H_42_O_9_	Roots	(32)
	94	Linderaggrenolide G	C_35_H_42_O_9_	Roots	(32)
	95	Linderaggrenolide H	C_30_H_37_ClO_7_	Roots	(32)
	96	Linderaggrenolide I	C_30_H_37_ClO_7_	Roots	(32)
	97	Linderaggrenolide J	C_31_H_40_O_8_	Roots	(32)
	98	Linderaggrenolide K	C_34_H_40_O_9_	Roots	(32)
	99	Linderaggrenolide L	C_34_H_40_O_9_	Roots	(32)
	100	Linderaggrenolide M	C_34_H_40_O_9_	Roots	(32)
	101	Linderaggrenolide N	C_31_H_34_O_8_	Roots	(32)
	102	Linderanoid A	C_29_H_30_O_6_	Roots	(33)
	103	Linderanoid B	C_33_H_34_O_8_	Roots	(33)
	104	Linderanoid C	C_29_H_32_O_6_	Roots	(33)
	105	Linderanoid D	C_30_H_34_O_4_	Roots	(33)
	106	Linderanoid E	C_30_H_34_O_5_	Roots	(33)
	107	Linderanoid F	C_30_H_34_O_6_	Roots	(33)
	108	Linderanoid G	C_30_H_34_O_6_	Roots	(33)
	109	Linderanoid H	C_30_H_34_O_3_	Roots	(33)
	110	Linderanoid I	C_30_H_34_O_5_	Roots	(33)
	111	Linderanoid J	C_30_H_34_O_6_	Roots	(33)
	112	Linderanoid K	C_29_H_34_O_4_	Roots	(33)
	113	Linderanoid L	C_30_H_34_O_5_	Roots	(33)
	114	Linderanoid M	C_32_H_36_O_6_	Roots	(33)
	115	Linderanoid N	C_32_H_36_O_7_	Roots	(33)
	116	Linderanoid O	C_34_H_38_O_8_	Roots	(33)
	117	Lindenaneolide F	C_30_H_34_O_6_	Roots	(33)
	118	Aggreganoid A	C_46_H_52_O_7_	Roots	(34)
	119	Aggreganoid B	C_46_H_52_O_6_	Roots	(34)
	120	Aggreganoid C	C_33_H_40_O_4_	Roots	(34)
	121	Aggreganoid D	C_31_H_34_O_6_	Roots	(34)
	122	Aggreganoid E	C_31_H_34_O_5_	Roots	(34)
	123	Aggreganoid F	C_31_H_36_O_5_	Roots	(34)
	124	Linderalide A	C_48_H_54_O_7_	Roots	(35)
	125	Linderalide B	C_48_H_54_O_6_	Roots	(35)
	126	Linderalide C	C_48_H_54_O_6_	Roots	(35)
	127	Linderalide D	C_49_H_58_O_7_	Roots	(35)

Sesquiterpene dimers are a characteristic class of constituents with C_30_ cores in *L. aggregata*, which are plausibly biosynthesized *via* the coupling of two identical or different sesquiterpenoid molecules (32). Forty-three dimers have been reported, among which lindenane-type sesquiterpenoid dimers are the most representative structures, such as linderaggrenolides A–N (88–101) and linderanoids H–O (109–116), where linderaggrenolides A–N have an oxygen bridge (32, 33). The remaining dimeric sesquiterpene components, such as linderanoids A–G (102–108), include 3 sesquiterpenoid dimers comprising a lindenane and a noreudesmane unit (102–104); 2 dimers consisting of a lindenane and an eudesmane unit (105–106), and 2 dimers containing a lindenane and an elemanolide unit (107–108) (33). Furthermore, 6 oligomeric sesquiterpenoids, aggreganoids A–F (118–123), were isolated from LA-R. Aggreganoid A and B (118–119) are 2 previously undiscovered methine- or methylene-bridged sesquiterpenoid trimers with a unique C_46_ skeleton. At the same time, aggreganoids C–F (120–123) are the first examples of carbon-bridged disesquiterpenoids with a C_33_ or C_31_ skeleton discovered in the plant kingdom (34). Linderalides A–C (124–126) are characterized by the unique disesquiterpenoid-geranylbenzofuranone hybrids directly linked by two C–C bonds. Linderalide D (127) possesses an unprecedented carbon skeleton with an unusual linearly 6/6/5/6/6 pentacyclic ring system fused by a sesquiterpenoid unit and a geranylbenzofuranone moiety (35). In addition, compound 86 shows moderate anti-coagulant activity, and compounds 95, 96, and 106 are excellent inhibitors against transforming growth factor-β (TGF-β) (19, 32, 33).

### 2.2. Alkaloids

**FIGURE 3:**
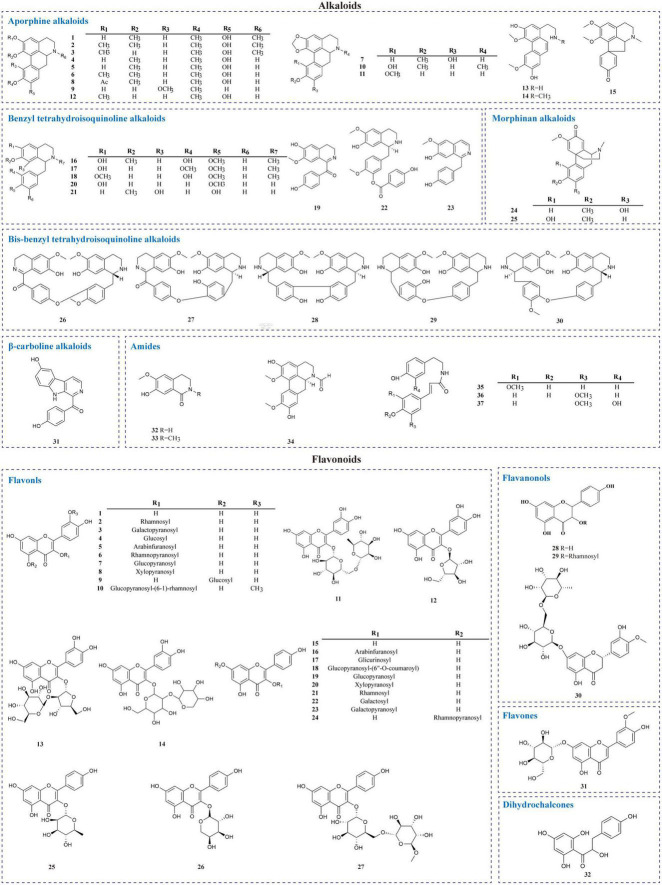
Chemical structures of alkaloids and flavonoids in *L. aggregata.*

Alkaloids are a massive group of naturally occurring organic compounds that contain one or more nitrogen atoms (amino or amide in some cases) in their structures. They are one of the active ingredients in *L. aggregata*, although the total content is not high (about 0.3%) (24). LA-R extract is the primary natural source of alkaloids. Thirty-seven alkaloids (1–37) have been successfully identified from *L. aggregata*, including 15 aporphine alkaloids (1–15), 8 benzyl tetrahydroisoquinoline alkaloids (16–23), 2 morphinan alkaloids (24, 25), 5 bis-benzyl tetrahydroisoquinoline alkaloids (26–30), a β-carboline alkaloid (31) and 6 amides (32–37) (13, 16, 24, 36–43). Their structures and molecular formulae are shown in Table 2 and Figure 3. Of these, isoquinoline alkaloids (1–30) account for a large proportion of the total alkaloids, and they have strong biological activities, such as anti-inflammatory (1–3, 12) (16, 37) and anti-cancer (5, 30) (37). Among them, argemexirine (20) is the first time to be isolated from Lauraceae (41). Apart from this, the only β-carboline alkaloid (31) found in LA-R displays significant potential against the superoxide anion generation (43).

**TABLE 2:** Alkaloids isolated from *L. aggregata.*

Subtype	No.	Constituents	Molecular formula	Parts	References
Aporphine alkaloids	1	Boldine	C_19_H_21_NO_4_	Roots	(36–38)
	2	(+)-*N-*methyllaurotetanine	C_20_H_23_NO_4_	Roots	(13)
	3	(+)-Isoboldine	C_19_H_21_NO_4_	Roots	(39)
	4	(+)-Norboldine	C_18_H_19_NO_4_	Roots	(13)
	5	Laurolitsine	C_18_H_19_NO_4_	Roots	(36, 37)
	6	(+)-Laurotetanine	C_19_H_21_NO_4_	Roots	(13)
	7	Actinodaphnine	C_18_H_17_NO_4_	Roots	(37)
	8	(+)-Norboldine acetate	C_20_H_21_NO_5_	Roots	(13)
	9	Linderaline	C_18_H_19_NO_4_	Roots	(36)
	10	(+)-Bulbocapnine	C_19_H_19_NO_4_	Roots	(24)
	11	Hernangerine	C_18_H_17_NO_4_	Roots	(40)
	12	Norisoboldine	C_18_H_19_NO_4_	Roots	(36, 37)
	13	Secolaurolitsine	C_18_H_19_NO_4_	Roots	(37)
	14	Secoboldine	C_19_H_21_NO_4_	Roots	(37)
	15	Pronuciferine	C_19_H_21_NO_3_	Roots	(36, 38)
Benzyl tetrahydroisoquinoline alkaloids	16	Protosinomenine	C_19_H_23_NO_4_	Roots	(36)
	17	Laudanosoline 3′,4′-dimethyl ether	C_19_H_23_NO_4_	Roots	(36)
	18	Reticuline	C_19_H_23_NO_4_	Roots	(36)
	19	Linderine A	C_17_H_15_NO_4_	Roots	(41)
	20	Argemexirine	C_17_H_19_NO_3_	Roots	(41)
	21	Norjuziphine	C_17_H_19_NO_3_	Roots	(37)
	22	(1*S*)-5′-*O*-*p*-hydroxy benzoyl norreticuline	C_25_H_25_NO_6_	Roots	(37)
	23	Yuzirine	C_17_H_15_NO_3_	Roots	(39)
Morphinan alkaloids	24	(−)-Pallidine	C_19_H_21_NO_4_	Roots	(36)
	25	Salutaridine	C_19_H_21_NO_4_	Roots	(37)
Bis-benzyl tetrahydroisoquinoline alkaloids	26	Linderegatine	C_35_H_34_N_2_O_7_	Roots	(37, 42)
	27	(1′*S*)-12′-hydroxyl-linderegatine	C_34_H_32_N_2_O_7_	Roots	(37)
	28	(1*R*,1′*R*)-11,11′-biscoclaurine	C_34_H_36_N_2_O_6_	Roots	(37)
	29	Lindoldhamine	C_34_H_36_N_2_O_6_	Roots	(37)
	30	Costaricine	C_35_H_38_N_2_O_6_	Roots	(37)
β -carboline alkaloid	31	Linderaggrine A	C_18_H_12_N_2_O_3_	Roots	(16, 43)
Amides	32	Northalifoline	C_10_H_11_NO_3_	Roots	(24, 39)
	33	Thalifoline	C_11_H_13_NO_3_	Roots	(39)
	34	Linderaggrine B	C_19_H_19_NO_5_	Whole plant	(16)
	35	*N-trans-*feruloyltyramine	C_18_H_19_NO_4_	Roots	(39)
	36	*N-cis-*feruloyltyramine	C_18_H_19_NO_4_	Roots	(39)
	37	*N-trans-*feruloylmethoxytyramine	C_18_H_19_NO_5_	Roots	(39)

### 2.3. Flavonoids

Flavonoids are one of the significant dietary polyphenols, mainly found in LA-L. Approximately 32 flavonoids and their glycosides have been isolated and identified (seen in Table 3 and Figure 3). The structural types include 27 flavonols (1–27), 3 flavanones (28–30), a flavone (31), and a dihydrochalcone (32) (4, 7, 18, 44–48). Among these, quercetin-3-*O*-β-D-glucoside (4), quercetin-3-*O*-β-D-arabinofuranoside (5), quercetin-3-*O*-α-L-rhamnopyranoside (QI) (6), quercetin-5-*O*-β-D-glucoside (9), kaempferol-7-*O*-α-L-rhamnopyranoside (24) were isolated and prepared by high-speed countercurrent chromatography (47). Furthermore, the total flavonoids extracted from LA-L have good antioxidant activity (49), and especially, QI (6) is an excellent antioxidant (50).

**TABLE 3:** Flavonoids isolated from *L. aggregata.*

Subtype	No.	Constituents	Molecular formula	Parts	References
Flavonols	1	Quercetin	C_15_H_10_O_7_	Leaves	(44)
	2	Quercetin-3-*O*-rhamnoside	C_21_H_20_O_11_	Leaves	(44)
	3	Quercetin-3-*O*-β-D-galactopyranoside	C_21_H_20_O_12_	Leaves	(44)
	4	Quercetin-3-*O*-β-D-glucoside	C_21_H_20_O_12_	Leaves	(47)
	5	Quercetin-3-*O*-β-D-arabinofuranoside	C_20_H_18_O_11_	Leaves	(47)
	6	Quercetin-3-*O*-α-L-rhamnopyranoside	C_21_H_20_O_11_	Leaves	(47)
	7	Quercetin-3-*O*-α-D-glucopyranoside	C_21_H_20_O_10_	Leaves	(46)
	8	Quercetin-3-*O*-β-D-xylopyranoside	C_20_H_18_O_11_	Leaves	(4)
	9	Quercetin-5-*O*-β-D-glucoside	C_21_H_20_O_12_	Leaves	(47)
	10	Isorhamnetin-3-*O*-[β-D-glucopyranosyl-(6→1)-rhamnoside]	C_28_H_32_O_16_	Leaves	(44)
	11	Rutin	C_27_H_30_O_16_	Leaves	(18)
	12	Avicularin	C_20_H_18_O_11_	Leaves	(45)
	13	Quercetin-3-*O*-(2″-*O*-β-D-glucopyranosyl)-α-L-arabinofuranoside	C_26_H_28_O_16_	Leaves	(7)
	14	Quercetin-3-*O*-(2″-*O*-β-D-glucopyranosyl)-β-D-xylopyranoside	C_26_H_28_O_16_	Leaves	(7)
	15	Kaempferol	C_15_H_10_O_6_	Leaves	(45)
	16	Kaempferol-3-*O*-L-arabinopyranoside	C_20_H_18_O_10_	Leaves	(44)
	17	Kaempferol-3-*O*-α-D-glucopyranoside	–	Leaves	(44)
	18	Kaempferol-3-*O*-(6″-*trans*-*p*-coumaroyl)-β-D-glucopyranoside	C_30_H_26_O_13_	Leaves	(18)
	19	Astragaline	C_21_H_20_O_11_	Leaves	(45)
	20	Kaempferol-3-*O*-β-D-xylopyranoside	C_20_H_18_O_10_	Leaves	(45)
	21	Kaempferol-3-*O*-L-rhamnoside	C_21_H_20_O_10_	Leaves	(46)
	22	Kaempferol-3-*O*-β-D-galactoside	C_21_H_20_O_11_	Leaves	(7)
	23	Kaempferol-3-*O*-β-D-galactopyranoside	C_21_H_20_O_11_	Leaves	(7)
	24	Kaempferol-7-*O*-α-L-rhamnopyranoside	C_21_H_20_O_11_	Leaves	(47)
	25	Afzelin	C_21_H_20_O_10_	Leaves	(45)
	26	Juglalin	C_20_H_18_O_10_	Leaves	(45)
	27	Kaempferol-3-*O*-(2″-*O*-β-D-glucopyranosyl)-α-L-rhamnopyranoside	C_27_H_30_O_16_	Leaves	(45)
Flavanones	28	Dihydrokaempferol	C_15_H_12_O_6_	Leaves	(45)
	29	Dihydrokaempferol-3-*O*-L-rhamnoside	C_21_H_20_O_10_	Leaves	(46)
	30	Hesperidin	C_28_H_34_O_15_	Roots	(48)
Flavone	31	Chrysoeriol-7-*O*-β-D-glucopyranoside	C_22_H_22_O_11_	Leaves	(18)
Dihydrochalcone	32	Nubigenol	C_15_H_14_O_6_	Leaves	(18)

### 2.4. Others

The current investigation on the phytochemicals obtained from *L. aggregata* is scarce, where 12 tannin-type components have been reported, including (+)-catechin (1), (−)-epigallocatechin (2), (−)-epicatechin (3), and diploid epicatechin-(4β-8, 2-*O*-7)-epicatechin (4), epicatechin-(4β-8, 2-*O*-7)-catechin (5), epicatechin-(4β-8)-catechin (6), procyanidin B_1_ (7) procyanidin B_2_ (8), and triploid aesculitannin B (9), cinnamtanin B_1_ (10), lindetannin trimer (12), and tetraploid Cinnamtannin B_2_ (11). In addition, procyanidin B_2_ (8) and aesculitannin B (9) are separated from the roots (48, 51–53). Additionally, epicatechin (3) and aesculitannin B (9) are non-competitive inhibitors against prolyl endopeptidase from *Flavobacterium meningosepticum*, and these three compounds (7, 10–11) have inhibitory activities against HIV-1 integrase (52, 53).

**FIGURE 4:**
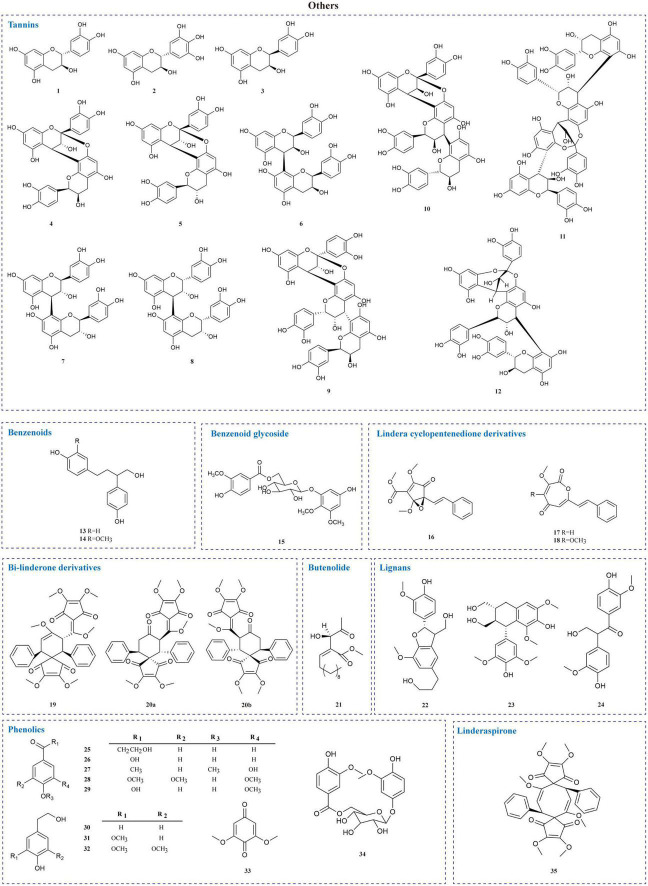
Chemical structures of other components in *L. aggregata.*

In addition to the above-mentioned extensive components in *L. aggregata*, there are also several other rare components, including 2 benzenoids (13–14), a benzene-type glycoside (15), 3 lindera cyclopentenedione derivatives (16–18), 3 bi-linderone derivatives (19, 20a, 20b), a butenolide (21), 3 lignans (22–24), 10 phenolics (25–34) and a linderaspirone (35) (16, 39, 54, 55). They also demonstrate good pharmacological activities, such as compound 21 has anti-tumor activity, and compounds 19 and 35 show good anti-diabetic properties due to their significant insulin resistance alleviation (55–57). The isolated phytochemicals are tabulated in Table 4 and Figure 4.

**TABLE 4:** Other components isolated from *L. aggregata.*

Subtype	No.	Constituents	Molecular formula	Parts	References
Tannins	1	(+)-catechin	C_15_H_14_O_6_	Stems	(51)
	2	(−)-epigallocatechin	C_15_H_14_O_7_	Stems and roots	(51)
	3	(−)-epicatechin	C_15_H_14_O_6_	Stems and roots	(51)
	4	Epicatechin-(4β-8,2-*O*-7)-epicatechin	C_30_H_24_O_12_	Stems	(51)
	5	Epicatechin-(4β-8,2-*O*-7)-catechin	C_30_H_24_O_12_	Stems	(51)
	6	Epicatechin-(4β-8)-catechin	C_30_H_26_O_12_	Stems	(51)
	7	Procyanidin B_1_	C_30_H_26_O_12_	Stems	(52)
	8	Procyanidin B_2_	C_30_H_26_O_12_	Roots	(48)
	9	Aesculitannin B	C_45_H_36_O_18_	Roots	(53)
	10	Cinnamtannin B_1_	C_45_H_36_O_18_	Stems and roots	(52)
	11	Cinnamtannin B_2_	C_60_H_48_O_24_	Stems	(52)
	12	Lindetannin trimer	C_45_H_36_O_18_	Stems	(52)
Benzenoids	13	Linderagatin-A	C_16_H_18_O_3_	Roots	(39)
	14	Linderagatin-B	C_17_H_20_O_6_	Roots	(39)
Benzenoid glycoside	15	6′-*O*-vanilloyl-5-hydroxy-2,3-dimethoxyphenol 1*-O*-β-D-glucopyranoside	C_22_H_26_O_12_	Whole plants	(16)
Lindera cyclopentenedione derivatives	16	(±)-lindepentone A	C_17_H_16_O_4_	Roots	(54)
	17	Lindoxepine A	C_15_H_12_O_4_	Roots	(54)
	18	Lindoxepine B	C_16_H_14_O_5_	Roots	(54)
Bi-linderone derivatives	19	Bi-linderone	C_34_H_32_O_10_	Roots	(57)
	20a	(+)-demethoxy-*epi*-bi-linderone	C_33_H_30_O_10_	Roots	(54)
	20b	(−)-demethoxy-*epi*-bi-linderone	C_33_H_30_O_10_	Roots	(54)
Butenolide	21	Secoaggregatalactone A	C_17_H_30_O_4_	Leaves	(55)
Lignans	22	Rel-(2α,3β)-7-*O*-methylcedrusin	C_20_H_24_O_6_	Roots	(39)
	23	(−)-Lyoniresinol	C_22_H_28_O_8_	Roots	(39)
	24	Evofolin B	C_17_H_18_O_6_	Roots	(39)
Phenolics	25	3-hydroxy-1-(4-hydroxyphenyl)propan-1-one	C_9_H_10_O_3_	Roots	(39)
	26	*p*-hydroxybenzoic acid	C_7_H_6_O_3_	Roots	(39)
	27	4-hydroxy-3-methoxy acetophenone	C_9_H_10_O_3_	Roots	(39)
	28	Methyl 3,5-dimethoxy-4-hydroxybenzoate	C_10_H_12_O_5_	Roots	(39)
	29	Vanillic acid	C_8_H_8_O_4_	Roots	(39)
	30	Tyrosol	C_8_H_10_O_2_	Roots	(39)
	31	2-(4-hydroxy-3-methoxyphenyl)-ethanol	C_9_H_12_O_3_	Roots	(39)
	32	2-(4-hydroxy-3,5-dimethoxyphenol)-ethanol	C_10_H_14_O_4_	Roots	(39)
	33	2,6-dimethoxy-*p*-benzoquinone	C_8_H_8_O_4_	Roots	(39)
	34	6′-*O*-vanilloyltachioside	C_21_H_24_O_11_	Roots	(39)
Linderaspirone	35	(±)-Linderaspirone A	C_34_H_32_O_10_	Roots	(56)

### 2.5. Essential oils

Essential oils are found in different parts of *L. aggregata*, including leaves, seeds, peels, tubers, and taproots. The composition of essential oils from *L. aggregata* is very complex, mainly composed of monoterpenes and sesquiterpenes, which usually exist in the form of oxygen-containing derivatives (alcohols, ketones, and lactones) and hydrocarbons, and GC-MS analysis is generally used to identify various essential oils from *L. aggregata* (58). Due to the distinctive bioactivities of sesquiterpenes, it has been listed separately in Section “2.1. Sesquiterpenoids” for a more explicit demonstration. They have important physiological and biological activities, such as anti-bacterial and anti-cancer activities (59, 60). Meanwhile, researchers have shown that the main components and contents of the essential oils from LA-R and LA-L are obviously different. The major compounds of the leaf oil are sesquithuriferol (35.90%), 14-oxy-α-muurolene (16.45%), etc. In comparison, the root oil is rich in zerumbone (26.66%), geranyl acetate (12.45%), (*E*)-β-ocimene (10.27%) (60). Due to different geographic origins, growing years, and/or harvest times, another research report showed that the main components of leaf oil were curzerene (12.60%), 1,4-diethyl-benzene (11.01%), and 2-methyl-6-(2-propenyl)-phenol (10.25%), while roots oil was rich in linderene (39.44%) and lindenenol (20.93%) (61). The phytochemical research on different parts of *L. aggregata* further laid the foundation for developing and utilizing its whole plant resources.

## 3. Pharmacology

**FIGURE 5:**
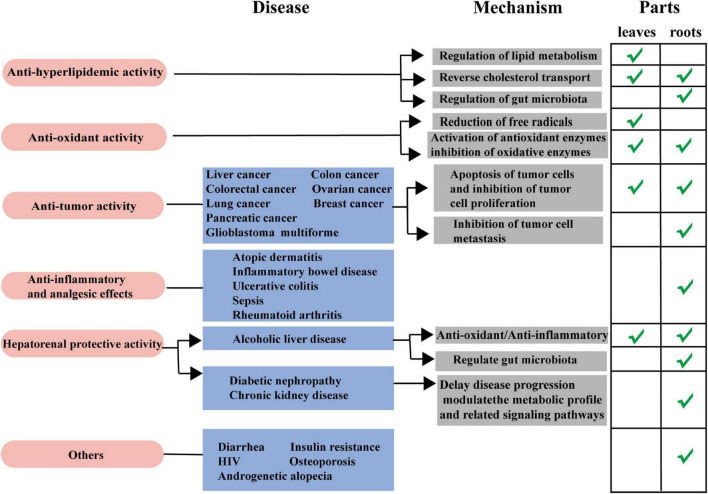
Schematic diagram of pharmacological activities from the roots and leaves of *L. aggregata.*

As a traditional medicine and edible plant, *L. aggregata* has been reported with multiple pharmacological activities and health functions based on *in vitro* and *in vivo* studies, including anti-hyperlipidemic, anti-oxidant, anti-tumor, anti-inflammatory, hepatoprotective, deworming, etc. Reports on the pharmacological activity of *L. aggregata* mainly focus on its crude extracts, while reports on active compounds mainly focus on flavonoids, alkaloids, sesquiterpenes, and essential oils. In the following parts, the primary pharmacological activities, health functions, and related molecular mechanisms of the crude extracts of *L. aggregata* and its bioactive compounds are summarized and discussed in detail, as illustrated in Figure 5 and Table 5.

**TABLE 5:** Pharmacological activities of different parts of *L. aggregata*.

Crude drugs/Compounds	Study type	Model method	Dose range/Concentration	Main effects	References
**Anti-hyperlipidemic activity**					
Aqueous extract of *L. aggregate* leaves	*In vivo*	HLP model of SD rats	0.33, 0.66, and 2.00 g/⋅bw (i.g., for 45 days)	TG↓, TC↓, LDL-C↓ HDL-C/LDL-C↑, HDL-C/TC↑, body ratio↓, liver weight↓, liver ratio↓	(63)
Extract of *L. aggregate* leaves	*In vivo*	HLP model of SD rats	0.8 and 1.6 g/kg (i.g., for 8 weeks)	TG↓, TC↓, LDL-C↓, AMPKα protein phosphorylation↑	(64)
Aqueous extract of *L. aggregate* leaves	*In vivo*	HCL mice	0.3, 0.6, and 1.2 g/kg (i.g., for 10 days)	Serum TC↓, TG↓, LDL↓, non-HDL↓, ALT↓, GLU↓, Apolipoprotein B↓, hepatic GLU↓, serum HDL↑, apolipoprotein A1↑, fecal TG levels↑	(66)
Ethanol extract of *L. aggregate* roots	*In vivo*	HLP model of SD rats	1, 2, and 4 g/kg (i.g., for 5 weeks)	TG↓, TC↓, LDL-C↓, AST activity↓, HDL-C↑	(67)
Ethanol extract of *L. aggregate* roots	*In vivo*	HLP model of SD rats	1 g/kg (i.g., for 3 weeks)	Intestinal microbiota diversity↑, bile acid reabsorption↑	(69)
**Anti-oxidant activity**					
Aqueous extract of *L. aggregate* leaves	*Clinical trial*	Young, healthy men	3 bags/day	Free radicals generated during the oxidative denaturation of LDL↓	(71)
Quercetin-3-*O*-α-L-rhamnopyranoside from *L. aggregate* leaves	*In vitro*	Oxidative stress model in HUVEcs	0, 62.5, 125, 250, and 500 μM	SOD↑, glutathione↑, caspase-9↓, poly (AdP-ribose) polymerase↓, MDA↓, mitochondrial SOD2↓	(50)
Lindenenyl acetate from *L. aggregate* roots	*In vitro*	Mouse hippocampal HT22 cells	10, 20, 30, and 40 μM	HO-1 expression↓, extracellular regulated protein kinases↑	(27)
**Anti-tumor activity**					
Aqueous extract of *L. aggregate* roots	*In vitro* and *in vivo*	Two human lung cancer cell lines (SBC-3, A549); C57BL/6 mice and BALB/c nu/nu nude mice	250 μg/mL for 48 h; 5 mg/kg (i.g., for 2 months)	Tumor growth↓	(73)
Isolinderalactone from *L. aggregate* roots	*In vitro*	SKOV-3, OVCAR-3 cells	0, 5, 10, 20, and 50 μM for 24 h	Mitochondrial superoxide↑, mitochondrial SOD2↓, Cell proliferation↓	(29)
Isolinderalactone from *L. aggregate* roots	*In vitro*	Human breast cancer cells MDA-MB-231	1, 10, and 20 μM for 24 h	STAT3 activation↓, cytokine signaling 3↑, Cell proliferation↓	(28)
Isolinderalactone from *L. aggregate* roots	*In vitro* and *in vivo*	Human U-87 glioblastoma cell line	1, 2.5, and 5 mg/kg every other day	Cell proliferation↓	(30)
Isolinderalactone from *L. aggregate* roots	*In vitro*	Human CRC ox-sensitive and ox-resistant cells	3, 6, and 9 μM for 24–48 h	Cell proliferation↓	(31)
Isolinderalactone from *L. aggregate* roots	*In vitro*	Human lung cancer A549 cells	1–10 μM for 24–48 h	Cell invasion↓, cell migration↓	(76)
3-oxo-5 aH,8bH-eudesma1,4(15),7(11)-trien-8,12-olide, 3-oxo4,5aH,8bH-eudesma-1,7(11)-dien-8,12-olide from *L. aggregate* roots	*In vitro*	Human small cell lung cancer cell (SBC-3)	–	IC_50_ = 7.2 and 32.2 μM	(15)
Linderalactone from *L. aggregate* roots	*In vitro*	Pancreatic cancer cell lines (ASPC-1, BXPC-3, CFPAC-1, and SW-1990)	0, 30, 40, 50, 60, 70, 80, 90, and 100 μM for 24–48 h	Cell proliferation↓	(74)
Essential oil from *L. aggregate* leaves	*In vitro*	A549, HeLa, Hep G2, and HUVEC	12.5–400 μg/mL for 24 h	IC_50_ = 22–24 μg/mL	(60)
Essential oil from *L. aggregate* roots	*In vitro*	Human esophageal cancer Eca-109 and human gastric cancer SGC-7901 cell lines	6.25, 12.5, 25, 50, 100, 200, and 400 μg/mL for 24 h	IC_50_ = 24.8 μg/mL	(75)
Secoaggregatalactone A from *L. aggregate* leaves	*In vitro*	Hep G2 cell line	4, 7, and 10 μg/mL; 13.8, 23.4, and 33.4 μM for 24 h	EC_50_ = 6.61 μg/mL; 22.1 μM	(55)
Costaricine, laurolitsine from *L. aggregate* roots	*In vitro*	Human colon carcinoma cell line (HCT-116)	–	IC_50_ = 51.4 and 27.1 μM	(37)
**Anti-inflammatory and analgesic effects**					
Ethanol extract of *L. aggregate* roots	*In vivo*	Colitis model mice	0.5, 1, and 2 g/kg (i.g., for 14 days)	IL-6↓, the signal transduction of IL-6/STAT3 signaling pathway↓, Th17 cells↓	(77)
Total alkaloids of *L. aggregata* roots	*In vivo*	Kunming mice induced with *p*-xylene and carrageenan and hot plate test and acetic acid writhing method	0.2 mL/10 g (i.g., for 5 days)	The pain threshold↑, times of twisting body↓, times of licking hind↓	(79)
Total alkaloids of *L. aggregata* roots	*In vivo*	CIA mice	50, 100, and 200 mg/kg (i.g., for 20 days)	The serum level of anti-CII IgG↓, lymphocyte proliferation↓	(80)
Total alkaloids of *L. aggregata* roots	*In vitro*	RAW 264.7 cells	10, 30, 100, and 300 μg/mL for 20 h	TNF-α↓, IL-1β↓, inducible NO synthase↓	(81)
Norisoboldine from *L. aggregate* roots	*In vivo*	CIA rats	10, 20, 40 mg/kg (i.g., for 20 days)	The swelling of paws and arthritis index scores↓, the infiltration of inflammatory cells↓, synovial hyperplasia↓, the serum level of anti-CII IgG↓, lymphocyte proliferation↓	(83)
Norisoboldine from *L. aggregate* roots	*In vitro*	FLS from CIA rats	10, 30, and 60 μM for 20 h	IL-6↓, MAPKs↓, PKC↓, NF-κB-p65↓, cAMP response element-binding protein↓	(84)
Norisoboldine from *L. aggregate* roots	*In vitro*	RAW264.7 cells	10 and 30 μM for 20 h	Osteoclast differentiation↓, the expressions of the bone matrix-degrading enzymes↓	(85)
Norisoboldine from *L. aggregate* roots	*In vitro*	FLS from adjuvant-induced arthritis rats	10, 30, and 100 μM for 24 h	Caspase 3↑, caspase 9↑, apoptosis rate↓, the cleavage of poly (ADP-ribose) polymerase	(86)
Norisoboldine from *L. aggregate* roots	*In vitro*	Synovium tissues from adjuvant-induced arthritis rats	1, 3, 10, and 30 μmol for 24 h	The number of blood vessels↓, the expression of growth factors in the synovium↓	(88)
Norisoboldine from *L. aggregate* roots	*In vitro*	HUVECs	1, 3, 10, 30, 60, and 100 μM for 24 h	Inhibit VEGF-induced endothelial cell migration	(87)
Norisoboldine from *L. aggregate* roots	*In vitro*	RAW264.7 cells	3, 10, and 30 μM for 24 h	Osteoclast differentiation↓, bone erosion↓	(89)
Norisoboldine from *L. aggregate* roots	*In vivo*	CIA rats	15 and 30 mg/kg (i.g., for 14 days)	The expression of Foxp3 mRNA in both gut and joints↑; the number of integrin α4β7 (a marker of gut source)-positive Foxp3+ cells in the joints↑	(90)
Norisoboldine from *L. aggregate* roots	*In vivo*	CIA rats	40 mg/kg (i.g., for 14 days)	Treg cells↑, Th17 cells↓	(91)
Norisoboldine from *L. aggregate* roots	*In vitro* and *in vivo*	K562-luc cells, DNCB-induced dermatitis model	2–50 μM; 10 mg/kg (i.p. for 22 days).	Inhibits NFAT activation, atopic dermatitis-like inflammatory reaction↓	(92)
Norisoboldine from *L. aggregate* roots	*In vitro* and *in vivo*	LPS-induced mice, RAW264.7 cells	10, 20, or 40 mg/kg for 24 h; 10, 20, and 40 μM	Regulate macrophage polarization	(93)
Norisoboldine from *L. aggregate* roots	*In vivo*	2,4,6-trinitrobenzenesulfonic acid-induced colitis mice model	20 and 40 mg/kg (i.g., for 7 days)	IL-1β↓, NLRP3↓, caspase-1	(94)
Norisoboldine from *L. aggregate* roots	*In vitro*	CD_4_^+^ T cells	1, 3, 10, and 30 μM	Treg cells↑	(95)
Norisoboldine from *L. aggregate* roots	*In vivo*	Dextran sulfate sodium salt-induced ulcerative colitis mice model	20 and 40 mg/kg (i.g., for 10 days)	β↓TNF-α↓, the activation of ERK, p38 MAPK and NF-κB-p65↑	(96)
Linderaggrine A from *L. aggregate* roots	*In vitro*	Human neutrophils	10 μM	IC_50_ = 9.17 ± 0.40 μM	(43)
Linderaggredin C (+)-N-methyllaurotetanine (+)-isoboldine from the whole plants of *L. aggregata*	*In vitro*	Human neutrophils	10 μM	IC_50_ = 7.45 ± 0.74, 8.36 ± 0.11, 5.81 ± 0.59 μM	(16)
Linderolide O, linderolide P from *L. aggregate* roots	*In vitro*	LPS-induced RAW264.7 macrophage cells	–	IC_50_ = 6.3, 9.6 μM	(20)
Norisoboldine and boldine from *L. aggregate* roots	*In vitro*	LPS-induced RAW264.7 macrophage cells	–	IC_50_ = 37.8, 38.7 μM	(37)
**Hepatorenal protective activity**					
Extract of *L. aggregate* roots	*In vivo*	Liver injury model SD rats	1 mL/100 g⋅bw (i.g., for 10 days)	The serum levels of ALT, AST, TG, TC, and MDA↓, the levels of MDA, NF-κB, TNF-α, and IL-1β in liver tissues↓	(100)
Total flavonoids of *L. aggregata* leaves	*In vivo*	The mice model of CCl_4_-induced acute liver injury	50–200 mg/kg (i.g., for 7 days)	ALT↓, AST↓, MDA↓, SOD activity, and total anti-oxidation capacity↑	(49)
Linderagalactone E, linderan, hydroxylindestenolide, and linderalactone from *L. aggregate* roots	*In vitro*	H_2_O_2_-induced oxidative damages on HepG2 cells	6.25, 12.5, 25, 50, 100, and 200 μM	EC_50_ = 67.5, 167.0, 42.4, and 98.0 μM	(13)
Ethanol extract of *L. aggregate* roots	*In vivo*	ALD model rats	1–4 g/kg (i.g., for 20 days)	ALT↓, AST↓, total bilirubin↓, IL-8↓, IL-6↓, NF-κB↓, TNF-α↓, LPS↓	(102)
Extract of *L. aggregate* roots	*In vivo*	ALD model rats	4 g/kg (i.g., for 33 days)	ALT↓, AST↓, total bilirubin↓, TNF-α↓, IL-6↓, IL-1β↓, LPS↓	(103)
Aqueous extract of *L. aggregate* roots	*In vivo*	C57BL/KsJ-*db/db* mice	730 mg/kg (i.g., for 12 weeks)	Glomerular sclerotic index↓, fibrosis in glomeruli↓, apoptotic rate of glomerular cells↓	(104)
Extract of *L. aggregate* roots	*In vivo*	Adenine-induced chronic kidney disease rats	0.75–3.52 g/kg (i.g., for 14 days)	Renal tubular dilatation↓, interstitial fibrosis↓, interstitial inflammation↓, modulate the metabolic profile and TGF-β/Smad signaling pathway	(98)

### 3.1. Anti-hyperlipidemic activity

**FIGURE 6:**
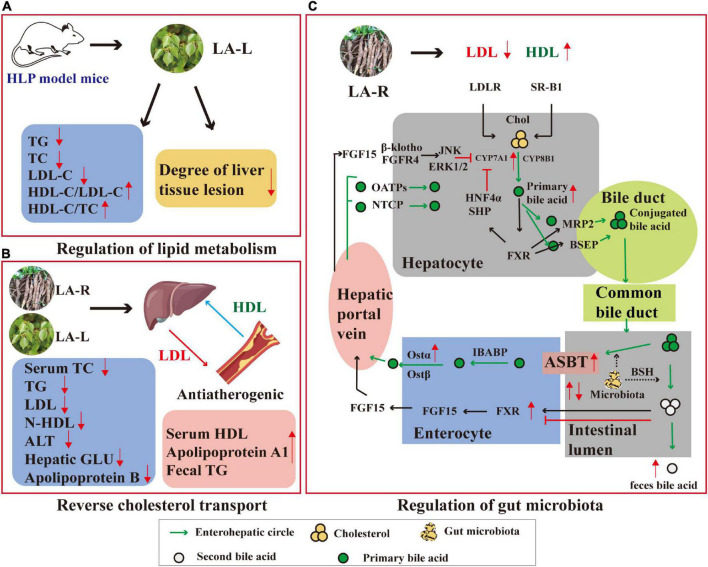
Anti-hyperlipidemic mechanism of action of *L. aggregata*. **(A)** Regulation of lipid metabolism. **(B)** Reverse cholesterol transport. **(C)** Regulation of gut microbiota. CYP8B1, Cytochrome P450, family 8, subfamily B, polypeptide 1; ERK, extracellular regulated protein kinases; FGF, recombinant fibroblast growth factor; β-klotho, a single-transmembrane receptor; OATPs and NTCP, drug transporters; MRP2 and BSEP, bile acid transporters; FXR, farnesoid X receptor; ASBT, apical sodium-dependent bile acid transporter; OST, organic solute transporter; BSH, bile salt hydrolase; HNF4α, hepatocyte nuclear factor 4α; SHP, small heterodimer partner.

Hyperlipidemia (HLP) is a common metabolic disorder, one of the principal positive risk factors for the development and progression of atherosclerosis and cardiovascular disease, caused by abnormal lipid metabolism or transport, and high levels of total cholesterol (TC), triglycerides (TG), low-density lipoproteins cholesterol (LDL-C), and high-density lipoprotein cholesterol (HDL-C). LA-R and LA-L have been proven to have good hypolipidemic effects (62). The hypolipidemic effects of *L. aggregata* are mediated by various mechanisms, including regulation of lipid metabolism, reverse cholesterol transport, and regulation of gut microbiota (Figure 6).

#### 3.1.1. Regulation of lipid metabolism

The crude extract of LA-L has demonstrated a specific lipid-lowering effect to improve HLP by promoting lipid metabolism. The aqueous extract of LA-L was used in HLP model mice, and blood lipids and histomorphology regulation were observed. The results showed that the aqueous extract of LA-L reduced TG, TC, and LDL-C levels while increasing HDL-C/LDL-C and HDL-C/TC compared with the control group. In the meantime, it relieved liver injury, cell swelling, degeneration, and other lesions in HLP model mice (63). In addition, further by observing the degree of lipid accumulation and detecting the expression of liver kinase B1 (LKB1)-adenosine 5′-monophosphate-activated protein kinase (AMPK) pathway-related proteins in HLP model mice liver, it was found that the possible action mechanism of promoting lipid metabolisms is the activation of AMPKα protein phosphorylation (64).

#### 3.1.2. Reverse cholesterol transport

Reverse cholesterol transport describes HDL’s metabolism and a crucial antiatherogenic function (65). The aqueous extract of LA-L was probed using hypercholesterolemic (HCL) mice for anti-hyperlipidemic effects and potential mechanisms. The outcomes presented that the aqueous extract of LA-L (0.3, 0.6, and 1.2 g/kg) significantly lowered serum TC, TG, LDL, non-HDL, alanine aminotransferase (ALT), hepatic lipid/glucose (GLU), apolipoprotein B, hepatic GLU, and increased serum HDL, apolipoprotein A1, and fecal TG levels in HCL mice. The potential cholesterol-lowering mechanism may involve inhibiting cholesterol synthesis by down-regulation of 3-hydroxy-3-methylglutaryl CoA reductase (HMGCR) and promoting cholesterol transport by up-regulation of cholesterol 7-α-hydroxylase (CYP7A1) and ATP-binding cassette transporter A1 (ABCA1) (66).

Besides, the ethanol extract of LA-R also encourages the conversion of cholesterol to the liver and bile acids, which may be associated with reverse cholesterol transport regulation to restore the abnormalities of bile acid metabolism caused by HLP (67).

#### 3.1.3. Regulation of gut microbiota

According to recent research, HLP is closely related to gut microbiota and high-fat diet (68). Research has shown that the ethanol extract of LA-R improved gut microbiota disturbance caused by a high-fat diet *via* increasing intestinal microbiota diversity and changing the abundance of the firmicutes, bacteroidetes, and actinobacteria. In addition, the ethanol extract of LA-R can increase bile acid reabsorption and promote fecal excretion through farnesoid X receptor, apical sodium-dependent bile acid transporter, organic solute transporter α, and CYP7A1 thus restoring abnormal bile acid metabolism caused by HLP (69).

### 3.2. Anti-oxidant activity

Oxidative stress is reflected in various diseases, including cancer, cardiovascular diseases, neurodegenerative diseases, diabetes, ischemia/reperfusion injuries, rheumatoid arthritis, and even the process of aging. So it is increasingly important to explore natural anti-oxidants that can mop up reactive oxygen species that trigger the development of chronic diseases (70).

#### 3.2.1. Reduction of free radicals

*Lindera aggregata* is a good source of natural antioxidants. As an edible plant, LA-L is consumed as a functional tea in Japan (Xufu Tea). The study showed that the generation of free radicals was significantly reduced during the oxidative denaturation of LDL after young and healthy men drank the tea for a week, which confirms the significant anti-oxidant activity of Xufu Tea in a healthy human environment (71).

#### 3.2.2. Activation of antioxidant enzymes and inhibition of oxidative enzymes

Flavonoids are the main bioactive ingredients responsible for the anti-oxidant effect of *L. aggregata*. It is noteworthy that flavonoids are rich in LA-L. In mice model with CCl_4_-induced acute liver injury, LA-L-flavonoids significantly decreased the ALT and aspartate aminotransferase (AST) activities and malondialdehyde (MDA) content while also significantly increasing the activities of superoxide dismutase (SOD) and total anti-oxidation capacity in serum at the concentrations of 50–200 mg/kg. Furthermore, the mRNA expression of thioredoxin, heme oxygenase (HO)-1, and peroxiredoxin-1 in liver tissues was also increased by LA-L-flavonoids (49).

**FIGURE 7:**
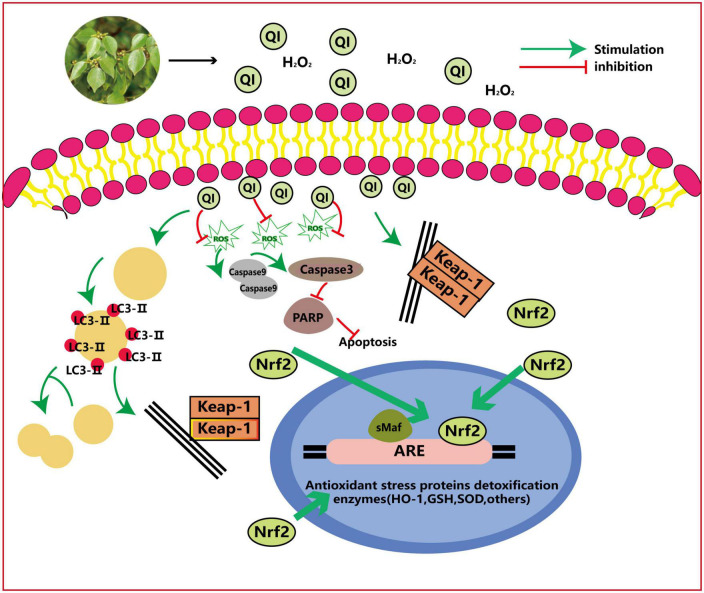
Anti-oxidant mechanism of action of QI: QI induces major cellular anti-oxidant enzymes to trigger autophagy *via* Nrf2 activation and Nrf2-dependent and effectively attenuates H_2_O_2_-induced oxidative stress in HUVEcs. ROS, reactive oxygen species; Lc, light chain; Keap1, Kelch-like EcH-associated protein 1; PARP, poly (AdP-ribose) polymerase; ARE, antioxidant response element; GSH, glutathione.

QI (compound 6 in Table 3) is the most representative flavonoid typically extracted from the leaves and has excellent development and utilization value. The anti-oxidant activity of QI and its underlying molecular mechanism in human umbilical vein endothelial cells (HUVEcs) were investigated using hydrogen peroxide-induced HUVEcs and the aging rat model (Figure 7). QI could induce major cellular anti-oxidant enzymes to trigger autophagy by activating nuclear factor-E2-related factor 2 (Nrf2) activation and Nrf2-dependent, thus effectively attenuating H_2_O_2_-induced oxidative stress in HUVEcs (50, 72).

In addition to anti-oxidant activity in LA-L, another study reported that lindenenyl acetate (compound 23 in Table 1) isolated from the LA-R effectively prevented glutamate-induced oxidative damage. HO enzymes are essential components of the cellular anti-oxidant system, and Nrf2 can induce the expression of HO-1 and glutathione. In the mouse hippocampal HT22 cell line, when the concentration set from 10 to 40 μM, lindenenyl acetate dose-dependently increased HO-1 expression; when the concentration reached 20 μM, lindenenyl acetate dose-dependently increased HO activity. In addition, lindenenyl acetate resulted in nuclear aggregation of Nrf2 and increased the promoter activity of anti-oxidant response elements in the HT22 cell line (27).

### 3.3. Anti-tumor activity

The anti-tumor activity effects of extracts of *L. aggregata* have been widely investigated through a series of *in vivo* and *in vitro* experiments. Studies have uncovered that the fractions and ingredients isolated from LA-R and LA-L exerted wide-spectrum anti-tumor activity against liver cancer, colon cancer, lung cancer, colorectal cancer (CRC), ovarian cancer (OC), breast cancer, pancreatic cancer, and glioblastoma multiforme. Its anti-tumor action is generally attributed to suppressing tumor cell growth, affecting tumor cell apoptosis, autophagy, migration, and invasion processes (30).

#### 3.3.1. Apoptosis of tumor cells and inhibition of tumor cell proliferation

Different crude extracts of *L. aggregata* have been shown to have good anti-tumor activity. It is reported that aqueous extract of LA-R specifically inhibited the growth of lung cancer cell lines A549 (IC_50_: 250 μg/mL) and SBC-3 (IC_50_: 100 μg/mL). In addition, the extract with 5 mg/kg significantly suppressed the proliferation of the growth of lung cancer cells transplanted in C57BL/6 and BALB/c nude mice after 2 months of treatment (73). Essential oils from the LA-L showed cytotoxic activity against three cancer cell lines, A549, HeLa, and the human hepatocellular carcinomas (HepG2) cell line *in vitro* (IC_50_: 22–24 μg/mL) (60).

**FIGURE 8:**
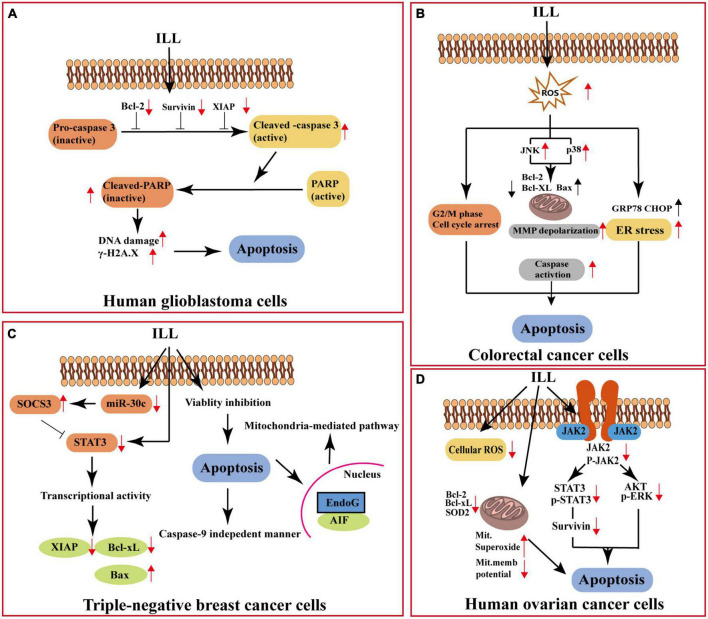
Anti-cancer mechanism of action of ILL. **(A)** Apoptosis of human glioblastoma cells. **(B)** Apoptosis of colorectal cancer cells. **(C)** Apoptosis of triple-negative breast cancer cells. **(D)** Apoptosis of human ovarian cancer cells. XIAP, X-linked inhibitor of apoptosis protein; PARP, poly (AdP-ribose) polymerase; ROS, reactive oxygen species; MMP, mitochondrial membrane potential; GRP78, 78-KDa glucose-regulated protein; CHOP, C/EBP homologous protein; SOCS3, cytokine signaling 3; miR-30c, microRNA hsa-miR30c-5p; AIF, apoptosis-inducing factor; EndoG, endonuclease G; JAK2, Janus kinase 2; ERK, extracellular regulated protein kinases.

The sesquiterpenoids of LA-R have been proven to possess good anti-tumor activity *in vitro* and *in vivo*. Among them, ILL (70 in Table 1) is the most representative, and it acts on different cancers through different pathways (Figure 8): it can induce apoptosis of human OC cells by increasing the production of mitochondrial superoxide, decreasing the expression of mitochondrial SOD2, and interfering with the signal transducer and activator of transcription 3 (STAT3)-mediated signaling pathway (29). In addition, ILL induced apoptosis of triple-negative breast cancer cells *via* suppressing STAT3 signaling pathway by regulation of suppressor of cytokine signaling 3 and micro-RNA 30c (28). And the expression levels of X-linked inhibitors of apoptosis and survivin in glioma cells were suppressed after treatment with ILL (30). It also demonstrated anti-tumor activity on CRC cells by inhibiting human CRC cell proliferation, inducing endoplasmic reticulum stress, modulating the G2/M phase of cell cycle progression, and inducing reactive oxygen species-mediated apoptosis through c-Jun N-terminal kinase (JNK)/p38 mitogen-activated protein kinases (MAPK) (31).

Other sesquiterpenoids from LA-R also have anti-tumor activity, such as 3-oxo-5aH,8bH-eudesma1,4(15),7(11)-trien-8,12-olide (compound 18 in Table 1), 3-oxo-4,5aH,8bH-eudesma-1,7(11)-dien-8,12-olide (compound 19 in Table 1) which showed potent cytotoxicity against human small cell lung cancer SBC-3 (IC_50_ values of 7.2, 32.2 μM), compared with positive control cisplatin (IC_50_ value of 8.6 μM) (15). Moreover, it was verified by *in vitro* and *in vivo* experiments that linderalactone (compound 53 in Table 1) inhibited the development of pancreatic cancer *via* negatively regulating the phosphatidylinositol 3 kinase (PI3K)/protein kinase B (AKT) signaling pathway (74). In another study by Yan et al. (75), the essential oils component germacrone showed obvious cytotoxicity to the proliferation of the seven human cancer cell lines tested, especially the inhibitory effect on the proliferation of human esophageal carcinoma Eca-109 and human gastric cancer SGC-7901 cell lines (IC_50_ = 24.8 μg/mL). Secoaggregatalactone A (compound 21 in Table 4), a seco butanolide from LA-L, exhibited noticeable cytotoxicity (EC_50_ of 6.61 μg/mL; 22.1 μM) against the HepG2 cell line (55). Furthermore, costaricine (compound 30 in Table 2) and laurolitsine (compound 5 in Table 2) from LA-R showed cytotoxic activities on the human colon carcinoma cell line (HCT-116), with IC_50_ values of 51.4 and 27.1 μM, respectively (37).

#### 3.3.2. Inhibition of tumor cell metastasis

ILL is a potential therapeutic adjuvant of A549 lung cancer cells and can inhibit the invasion and migration of A549 cancer cells. The author suggests that the possible mechanisms involve the inhibition of matrix metalloproteinase-2 and β-catenin protein expression resulting from the up-regulation of NME/NM23 nucleoside diphosphate kinase 1 expression (76).

### 3.4. Anti-inflammatory and analgesic effects

*Lindera aggregata* also exhibits anti-inflammatory properties, showing protective effects against inflammation-related diseases, such as rheumatoid arthritis, atopic dermatitis, inflammatory bowel disease, sepsis, and ulcerative colitis.

For the crude extract, the ethanol extract of LA-R diminished the production and secretion of interleukin (IL)-6, regulated IL-6/STAT3 signal transduction, and modulated the balance of T helper (Th) 17 and Treg cells to attenuate ulcerative colitis (77). The total alkaloids of LA-R have good analgesic and anti-inflammatory effects by observing the number of hind feet licking on a hot plate and times of twisting body in mice induced by acetic acid (78, 79). Using a model of collagen II (CII)-induced arthritis (CIA) rats, the study revealed that the total alkaloids of LA-R at 50, 100, and 200 mg/kg alleviated disease severity in a dose-dependent manner. Assessed by its effect on CII-induced ear swelling in mice, it also decreased serum levels of IgG anti-CII and inhibited delayed-type hypersensitivity at 100 and 200 mg/kg (80). The anti-inflammatory mechanism of the total alkaloids of LA-R may be through downregulating the functions of T lymphocytes and macrophages and nuclear factor kappa-B (NF-κB) and MAPKs signaling pathways (81).

Multiple studies have confirmed that the isoquinoline alkaloid norisoboldine (NOR) (compound 12 in Table 2) from LA-R is the most representative active compound and possesses outstanding anti-arthritis activity (82). In CIA rats, oral NOR (10, 20, and 40 mg/kg) significantly decreased the swelling of paws, arthritis index scores and elevated the lowered body weights of rats. It prevented the infiltration of inflammatory cells and the destruction of bone and cartilage in joints (83). NOR prevented the release of IL-6 from fibroblast-like synoviocytes (FLS), which may be related to the inhibition of protein kinase C (PKC)/MAPKs/NF-κB-p65/cAMP-response element binding protein (CREB) pathway (84). Another study by Wei et al. (85) showed that NOR was demonstrated to block osteoclast differentiation and function in the early stages of the TRAF6-TAK1 (a MAPK kinase) complex and to inhibit the resorptive function of osteoclasts by downregulating the expression of the bone matrix-degrading enzymes (cathepsin K and matrix metallopeptidase 9). The anti-arthritic mechanism of NOR may involve the inhibition of inflammatory synovial hyperplasia by promoting the release of cytochrome C and regulating the expression of B-cell lymphoma-2 (Bcl-2) and Bcl2-Associated X (Bax) proteins. Besides, some studies have shown that the inhibition of synovial angiogenesis and endothelial cell migration are also the contributed to anti-arthritic effect (86–88). NOR could also suppress osteoclast differentiation in rheumatoid arthritis and consequent joint bone impairment in an aryl hydrocarbon receptor-dependent manner (89). It can work by restoring systemic Th17/Treg balance *via* the induction of intestinal Treg cell generation and the migration of these cells to inflamed joints and synovium (90, 91).

Besides anti-arthritis activity, NOR has other activities. For example, NOR could reduce 2,4-dinitrofluorobenzene-induced dermatitis in mice by inhibiting nuclear factor of activated T-cells (NFAT) (92). It regulates the polarization of macrophages through the M2 pyruvate kinase (PKM2)/hypoxia-inducible factor (HIF-1α)/peroxisome proliferator activated receptor-γ co-activator 1-α (PGC-1α) pathway, thus alleviating sepsis-induced acute lung injury (93). It can also inhibit the activation of inflammatory bodies of protein 3 associated with nod-like receptor hot protein domain to weaken the colitis induced by 2,4,6-trinitrobenzene sulfonic acid in mice. Moreover, it ameliorated ulcerative colitis and alleviated the development of colitis in mice induced by dextran sodium sulfate by promoting the differentiation of Treg cells (94–96).

Other types of alkaloids and sesquiterpenoids also have good anti-inflammatory activity. For example, linderaggrine A (compound 31 in Table 2) (β-carboline alkaloid) showed significant anti-inflammatory activity in superoxide anion generation with an IC_50_ value of 9.17 ± 0.40 μM as compared to positive control sorafenib (IC_50_ = 3.23 ± 0.42 μM) (43). The sesquiterpenoid linderaggredin C (compound 83 in Table 1) and the alkaloids (+)-*N*-methyllaurotetanine (compound 2 in Table 2) and (+)-isoboldine (compound 3 in Table 2) were isolated from the whole plant of *L. aggregata*, displaying the significant inhibition of the generation of superoxide anion in human neutrophils with IC_50_ values of 7.45 ± 0.74, 8.36 ± 0.11, and 5.81 ± 0.59 μM, respectively (16). Inhibition of overstimulated inflammatory cytokines and NO is a potential therapeutic target for inflammatory disease. Linderolide O (compound 36 in Table 1), linderolide P (compound 37 in Table 1), NOR, and boldine (compound 1 in Table 2) inhibited lipopolysaccharide (LPS)-stimulated nitric oxide production in murine RAW 264.7 macrophage cells, with IC_50_ values of 6.3 and 9.6 37.8, and 38.7 μM, respectively (20, 37).

### 3.5. Hepatorenal protective activity

Liver and kidney injuries are common pathological processes. Liver injury can result in fatty liver, cirrhosis, fibrosis, and even cancer, while kidney injury can lead to arterial hypertension, proteinuria, hematuria, edema, etc. (97–99). According to records, *L. aggregata* is an excellent natural plant for protecting the liver and kidney. Numerous studies have investigated the hepatorenal protective effects of *L. aggregata* on alcoholic liver disease (ALD), diabetic nephropathy, and chronic kidney disease.

#### 3.5.1. Liver protection through anti-oxidants and anti-inflammatory activities

**FIGURE 9:**
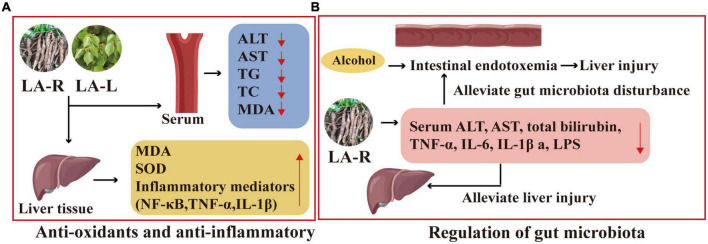
Liver protection mechanism of action of *L. aggregata*. **(A)** Anti-oxidants and anti-inflammatory. **(B)** Regulation of gut microbiota.

The LA-R extract may improve ALD through anti-oxidation and anti-inflammatory. Its experimental result includes improved histopathological status and reduced serum ALT, AST, TG, TC, and MDA levels; decreased MDA and inflammatory mediators, including NF-κB, tumor necrosis factor-α (TNF-α), and IL-1β in liver tissues; decreased ethanol treatment-induced overexpression of cytochrome P450 2E1 mRNA (100), as displayed in Figure 9.

LA-L-flavonoids (50–200 mg/kg) remarkably decreased ALT and AST activities and MDA concentration, and increased SOD activity and total anti-oxidation capacity in the serum of the mice model of CCl_4_-induced acute liver injury (49).

Linderagalactone E (compound 8 in Table 1), linderane (compound 54 in Table 1), hydroxylindestenolide (compound 3 in Table 1), and linderalactone from LA-R (EC_50_ values of 67.5, 167.0, 42.4, and 98.0 μM) have shown hepatoprotective activity against H_2_O_2_-induced oxidative damages on HepG2 cells. The mechanisms may be related to anti-oxidative stress as well as inhibiting the cytochrome P450 2E1 mRNA expression in rat liver (13).

#### 3.5.2. Liver protection through regulating gut microbiota

The prebiotic effect is essential for LA-R to attenuate the disturbance of gut microbiota in liver disease. Since intestinal permeability and intestinal endotoxemia caused by excessive alcohol consumption are the key pathogenic factors in the occurrence of ALD, improving intestinal function to relieve intestinal endotoxemia will be an effective method for the treatment of ALD (101).

Studies have shown that the ethanol extract of LA-R has a protective effect on the intestinal barrier and can alleviate gut microbiota disturbance, thereby reducing intestinal endotoxemia associated with alcoholic liver injury (102). Furthermore, compared with the ALD mouse model group, the 4 g/kg LA-R group inhibited alcohol-induced intestinal permeability by reducing serum ALT, AST, total bilirubin, TNF-α, IL-6, IL-1β, and LPS, which could alleviate liver injury (103), as indicated in Figure 9.

#### 3.5.3. Kidney protection through delaying disease progression, modulating the metabolic profile and related signaling pathways

The aqueous extract of LA-R (730 mg/kg/day) was orally administered to C57BL/KsJ-*db/db* mice to observe the progression of diabetic nephropathy. The results showed that it gradually worsened in the control group but remained unchanged in the treatment group, indicating that it can slow the progression of the disease and improve renal function (104). Another study found that LA-R attenuated adenine-induced chronic kidney disease mechanisms: modulation of metabolic profiles and TGF-β/Smad signaling (98).

### 3.6. Others

In addition to the bioactivities mentioned above, *L. aggregata* has also been observed other biological activities, such as insulin sensitivity, anti-osteoporosis (OP) effect, anti-viral activity, insecticidal activity, and anti-microbial activity. At a concentration of 1 μg/mL, bi-linderone (compound 19 in Table 4), (±)-linderaspirone A (compound 35 in Table 4) showed significant activity against glucosamine-induced insulin resistance in HepG2 cells (56, 57). Using a network pharmacology approach to explore the active components and underlying mechanisms of LA-R in OP treatment, the anti-OP activity of LA-R was validated in a prednisone-induced zebrafish model (105). Compared with the model group, the ethanol extract of LA-R significantly reduced the mRNA expression of both cathepsin K and acid phosphatase type 5a in zebrafish, indicating that it could significantly reduce osteoclast bone resorption by regulating the receptor activator of NF-κB/receptor activator of ligand/osteoclastogenesis inhibitory factor system and down-regulating cathepsin K and acid phosphatase type 5a (105). Oligomeric proanthocyanidins inhibited HIV-1 integrase with IC_50_ values ranging from 5.2 to 31.3 μM (52). The essential oils of *L. aggregate* was found to exhibit insecticidal activity against two-grain storage insects (*Sitophilus zeamais* and *Tribolium castaneum*) with LC_50_ values of 61.65 and 18.47 μg/adult (106). Apart from that, LA-R improves diarrhea and can treat androgenetic alopecia by controlling the scalp microbiome (107, 108).

## 4. Conclusion

*Lindera aggregata* has been widely used in traditional practices due to its potential efficacies for treating and preventing several diseases. This review provides a comprehensive investigation of the phytochemical constituents and pharmacological properties of *L. aggregata*. The primary chemical components isolated from the plant are sesquiterpenoids, alkaloids, and flavonoids. More importantly, they have shown interesting biological properties in various scientific investigations. Although a great deal of progress has been made in the study of *L. aggregata*, there are still some gaps and challenges in the findings of the existing research.

First of all, many studies have focused on validating the traditional pharmacological activities of crude extracts or a few unique chemical constituents. In contrast, the comprehensive phytochemical analysis of the assessed extract still needs to be improved, and the functional components still need to be discovered. As is known to all, the natural medicinal plant usually contains extremely complex phytochemical components. Different medicinal parts, such as LA-R and LA-L, contain various ingredients with distinctive skeletons. Different phytochemical profiles of herbs may result in various potencies in biological assessments. The synergistic effect of different components may also affect their pharmacological activities. Therefore, further development of phytochemical analysis is essential to determine the correlation between the components of different parts of *L. aggregata* and their pharmacological activities and to discover their promising precursors for health food or medicine.

As mentioned above, different parts of *L. aggregata* have different chemical compositions, which will affect its efficacy. The research cited in this review has focused on the tuberous roots and leaves of *L. aggregata*, and there is little research on the taproots. However, in the actual process, the mixing of taproot tubers and tuberous roots will lead to the quality decline of medicinal materials. The distinctions between these two types of roots of *L. aggregata* have been proved by comparing transcriptome, metabolome, and analgesic effects in 2020 (109). Simultaneously, in the latest research in the first half of 2022 (110), the portable short-wavelength infrared microscope hyperspectral imager combined with a machine learning algorithm was used to distinguish different types of roots (taproot tubers and tuberous roots) and different geographical origins of *L. aggregata* with high accuracy. Accordingly, future studies can focus on developing a more comprehensive, accurate, and convenient method to distinguish the taproots, tuberous roots, and the different origins to control the quality of *L. aggregata.*

Third, most of the above pharmacological studies have assessed pharmacological activity using simple *in vitro* cell lines or *in vivo* animal models, with only in-depth mechanism-of-action studies for a particular ingredient but no further investigation of potential mechanisms of action for most of the ingredients. Because the natural medicinal plant has the characteristics of multi-component and multi-target, further pharmacological research is needed to clarify it fully.

Fourth, there are few studies on the systemic toxicity of *L. aggregata*. As a medicinal and edible plant with development value, systematic safety assessment is crucial for evaluating the acute, chronic, reproductive, and genotoxicity of crude extracts and/or bioactive constituents in different experimental organisms.

Fifth, according to the unique components found in other plants of genus *Lindera*, such as phenylpropanoids, it is predicted that new components in *L. aggregata* may be further discovered.

Research *in vitro* and *in vivo* has been conducted, and the mechanism of action still needs to be in-depth. Still, the existing research lays the foundation for further exploration of new therapeutic uses of *L. aggregata.* As a new resource of functional food ingredients, the potential of LA-L in developing health products has attracted more and more attention, and its chemical composition and pharmacological research will continue to deepen.

## Author contributions

YL: conceptualization, writing, and editing of the manuscript. YZ and XZ: collection and compilation of information. BL, CC, and XP: conception, writing, review, and editing. All authors contributed to the article and approved the submitted version.
